# A Path FORWARD: Development of a Comprehensive Multidisciplinary Clinic to Create Health and Wellness for the Child and Adolescent with a Fontan Circulation

**DOI:** 10.1007/s00246-022-02930-z

**Published:** 2022-05-23

**Authors:** Jack Rychik, David J. Goldberg, Elizabeth Rand, Edna E. Mancilla, Jennifer Heimall, Nicholas Seivert, Danielle Campbell, Shannon O’Malley, Kathryn M. Dodds

**Affiliations:** 1 Division of Cardiology, Children’s Hospital of Philadelphia, Philadelphia, USA; 2 Division of Gastroenterology, Hepatology and Nutrition, Children’s Hospital of Philadelphia, Philadelphia, USA; 3 Division of Endocrinology and Diabetes, Children’s Hospital of Philadelphia, Philadelphia, USA; 4 Division of Allergy and Immunology, Children’s Hospital of Philadelphia, Philadelphia, USA; 5 Department of Child and Adolescent Psychiatry, and Behavioral Sciences, Children’s Hospital of Philadelphia, Philadelphia, USA; 6 Clinical Nutrition, Children’s Hospital of Philadelphia, Philadelphia, USA; 7 Department of Pediatrics, Perelman School of Medicine at the University of Pennsylvania, Philadelphia, USA; 8 Department of Psychiatry, Perelman School of Medicine at the University of Pennsylvania, Philadelphia, USA; 9 Department of Nursing at the University of Pennsylvania, Philadelphia, USA; 10 Fontan FORWARD Program, Cardiac Center at the Children’s Hospital of Philadelphia, 3401 Civic Center Boulevard, Philadelphia, PA 19104 USA

**Keywords:** Fontan circulation, Single ventricle, Fontan-associated liver disease, Multidisciplinary care

## Abstract

Today, it is anticipated most individuals diagnosed with single-ventricle malformation will survive surgical reconstruction through a successful Fontan operation. As greater numbers of patients survive, so has the recognition that individuals with Fontan circulation face a variety of challenges. The goal of a normal quality and duration of life will not be reached by all. The hurdles fall into a variety of domains. From a cardiovascular perspective, the Fontan circulation is fundamentally flawed by its inherent nature of creating a state of chronically elevated venous pressure and congestion, accompanied by a relatively low cardiac output. Ventricular dysfunction, atrioventricular valve regurgitation, and arrhythmia may directly impact cardiac performance and can progress with time. Problems are not limited to the cardiovascular system. Fontan circulatory physiology impacts a multitude of biological processes and health parameters outside the heart. The lymphatic circulation is under strain manifesting as variable degrees of protein-rich lymph loss and immune system dysregulation. Organ system dysfunction develops through altered perfusion profiles. Liver fibrosis is ubiquitous, and a process of systemic fibrogenesis in response to circulatory stressors may affect other organs as well. Somatic growth and development can be delayed. Behavioral and mental health problems are common, presenting as clinically important levels of anxiety and depression. Most striking is the high variability in prevalence and magnitude of these complications within the population, indicating the likelihood of additional factors enhancing or mitigating their emergence. We propose that optimal care for the individual with single ventricle and a Fontan circulation is ideally offered in a comprehensive multidisciplinary manner, with attention to elements that are beyond cardiac management alone. In this report, we share the concepts, our experiences, and perspectives on development of a clinic model—the “Fontan rehabilitation, wellness and resilience development” or FORWARD program. We provide insights into the mechanics of our multidisciplinary model of care and the benefits offered serving our growing population of individuals with a Fontan circulation and their families.

## Introduction

Single-ventricle forms of congenital heart disease are one of the most complex of all congenital heart malformations. Without care, these conditions are typically lethal. Over the past four decades, strategies have developed, which allow for a pathway of survival. A series of operations undertaken in the first few years of life assign the task of systemic blood flow to the single ventricle present, while pulmonary blood flow is achieved through staged cavopulmonary connection. The result is passive systemic venous return without pulsatile flow, a Fontan circulation.

Today, we can anticipate most individuals prenatally diagnosed with single-ventricle malformation will survive through a successful Fontan operation [1]. Having created a successful surgical strategy for single-ventricle birth defects is a milestone that cannot be under-estimated and should be considered a major achievement in the annals of modern cardiovascular care [2]. However, the current treatment is far from a cure. It is a palliative strategy. As greater numbers of individuals survive, so has the recognition that they face a variety of challenges. The goal of a normal quality and duration of life will not be reached by all.

The hurdles fall into a variety of domains [3]. From a cardiovascular perspective, the Fontan circulation is fundamentally flawed by its inherent nature of creating a state of chronically elevated venous pressure and congestion, accompanied by a relatively low cardiac output. Ventricular dysfunction, atrioventricular valve regurgitation, and arrhythmia may directly impact cardiac performance and can progress with time. Problems are not limited to the cardiovascular system. Fontan circulatory physiology impacts a multitude of biological processes and health parameters outside the heart. The lymphatic circulation is under strain manifesting as variable degrees of protein-rich lymph loss and immune system dysregulation. Organ system dysfunction develops through altered perfusion profiles. The organ most evidently impacted is the liver, with fibrosis common. A process of systemic fibrogenesis in response to circulatory stressors may affect other organs as well, as fibrosis can be seen in the kidneys and in the heart itself. Somatic growth and development can be delayed. Behavioral and mental health problems are common, presenting as clinically important levels of anxiety and depression. While these problems are now well recognized, their precise mechanistic origins are poorly understood. Most interesting is the strikingly high variability in prevalence and magnitude of these complications within the population, indicating the likelihood of additional factors enhancing or mitigating their emergence. Modifiers such as risk and resilience factors are likely at work but are yet unidentified.

It is our opinion that optimal care for the individual with single ventricle and a Fontan circulation is ideally offered in a comprehensive multidisciplinary manner, with attention to matters that are beyond cardiac management alone. Those with single ventricle and Fontan circulation are the farthest from a “cure” than are individuals with any other form of congenital heart disease and are, thus, most likely to benefit from such a comprehensive health-based approach. In this report, we share the concepts, our experiences, and perspectives on development of the “Fontan rehabilitation, wellness and resilience development” or FORWARD program at the Children’s Hospital of Philadelphia. We provide insights into the mechanics of our multidisciplinary model of care and the benefits offered serving our growing population of individuals with a Fontan circulation and their families. This report provides the community with insights on how to establish and replicate such a clinic, or a modification based on needs.

## Concept and Structure of the FORWARD Clinic

Over 10 years ago, we appreciated the pressing need to provide more than just cardiac care alone to our patients with Fontan circulation. Individuals were manifesting curious findings that led us to seek expertise outside of the cardiovascular realm for better understanding. Elevated liver enzymes might be coincidentally noted on routine blood laboratory testing, a distressing finding for both healthcare providers and families. Did this reflect development of circulatory failure requiring action, or was this an anticipated, typical phenomenon? Children would present with inexplicable lymphopenia. Was it a coincidence, or did many of our Fontan circulation children have a problem with viral warts and molluscum infection? Growth and pubertal delays were profound in some, but not present in others. Low serum albumin levels with mild peripheral swelling might present transiently after a viral process, only to spontaneously resolve. Children from stable families were exhibiting clinically significant anxiety, behavioral disturbances, and executive dysfunction. Were these phenomena and others random, or was there a connection to single ventricle and Fontan circulation?

The surgical strategy of care for single ventricle provides for the emergence of a human population with an unnatural circulation, individuals who have “never before walked the face of the earth.” Acknowledging this uniqueness creates an obligation to better understand how and why these individuals are different in greater detail. In 2011, we gathered a team of healthcare providers consisting of experts in cardiology, hepatology, endocrinology, and clinical immunology under the coordination of an experienced nurse practitioner and created a multidisciplinary clinic to develop a systematic approach and focus attention on these problems.

## How Did We Start?

### Developing Expertise

Pediatric sub-specialty expertise exists at many excellent centers. However, even among the most brilliant minds in the fields of pediatric hepatology, immunology, or endocrinology, we realized there was little experience in caring for the needs of a child with a Fontan circulation outside of cardiovascular specialists. Expertise needed to be developed and concentrated within a dedicated group of individuals. First, we selectively identified professionals who were clinically experienced in their own fields, but also curious about this unique population. Distributing our Fontan circulation patients to a wide number of pediatric sub-specialists would dilute the individual provider experience and counter the development of expertise. Thus, building a small team of sub-specialists, comfortable with understanding Fontan circulatory physiology and facile with the language, was the goal. This demanded a commitment of time and energy on the part of busy pediatric sub-specialists to focus their attention on this population, beyond their other duties. An attraction was the opportunity for academic productivity, as participation in this effort could provide great potential for new areas of clinical research and publication as well as the satisfaction of providing care for a unique population. Sub-specialty divisional leaders, department chairs, and hospital administrators were engaged and persuaded of the value of such a multidisciplinary endeavor and were instrumental in allowing sub-specialist participation and providing institutional support.

Next, we needed to grow a large cohort of patients and build the exposure for the team to develop expertise. The model we chose was that of a consultation service. Patients and families would continue ties to their primary cardiologists, as personal close relationships have developed over the years of care, often starting in fetal or newborn life. There was no interest in interrupting the important patient–provider dynamic that exists. We focused on how patients could be referred to our clinic as an *additional layer of care*, with added value on top of their primary cardiology services. Our multidisciplinary clinic would provide a consultative “deeper dive” into non-cardiac domains and offer the expertise of interpretation of characteristics discovered upon standardized clinical surveillance investigations. Detailed summaries of our characterization profile would be provided to families and to the primary cardiologists for analysis and review. Recommendations would be made, with rationale provided, but executed only under collaborative approval with the referring cardiologist. The goal was to create a favorable dynamic where the clinic would not be perceived as competing or interfering with primary cardiology care, but rather as a complimentary service. Demonstrating incremental added value to patients and providers was essential.

Financial investment was necessary up front to start the program at first, primarily to support a dedicated clinical nurse coordinator and administrative efforts for patient reception, communication, and scheduling. Pediatric sub-specialist support of time and effort on the part of various participating practitioners was necessary from departmental and divisional leaders. However, it became abundantly clear rather quickly that the detailed evaluations required by the program engendered clinical encounters, testing, and assessments—for both cardiac and non-cardiac clinical domains—that would not have otherwise been performed and, thus, generated new streams of institutional and clinical practice revenue.

### Foundational Philosophy

The clinical approach adopted is to learn as we go. Complete solutions to complex problems may not yet be evident. Exploration and discovery are necessary, but at the same time, certain things can be done and actions can be taken to improve clinical status and outcomes. Early in our experience, there was wide concern expressed that a focus on non-cardiovascular matters might create unnecessary anxiety among patients and families. Some cardiology practitioners were of the view that focusing on non-cardiac matters was “creating problems that did not exist” and “frightening the patients.” Interestingly, shortly after establishment of the clinic, feedback from families was overwhelmingly positive. There was an understanding of the importance of these efforts and families commonly expressed relief that as a provider community, we were finally focusing attention on the overall comprehensive health of children with a Fontan circulation.

The general philosophy is that a proactive approach is essential. We aim to characterize problems and apply general principles of diagnostics and care to improve outcomes. Low or subclinical levels of conditions may be evident upon investigation. The earliest detection and recognition allow the opportunity for serial monitoring, and often, therapeutic intervention, which may stave off more serious complications over time.

### Evolution of the Program

The original endeavor was called the Children’s Hospital of Philadelphia (CHOP) “Single Ventricle Survivorship Program” modeled after the concept of a cancer survivorship clinic [4]. Similarities between cancer survivorship and Fontan survivorship do exist, with the notion that once “treated” there are still life-long manifestations that require attention. However, over time we appreciated the need to evolve the concept of care in a different direction and that there were differences between cancer and single-ventricle survivorship. Primarily, while injury from therapy is potentially present in both cancer and single-ventricle treatments, those with Fontan circulation manifest unique, often new, active, and ongoing consequences of an altered circulatory state that are sustained in a continuous manner, long after operation. Furthermore, feedback provided by families encouraged us to move away from the use of “survivorship” and towards a more optimistic and positive language, actively promoting wellness. In 2017, the program evolved, and we established the “FORWARD”—Fontan rehabilitation, wellness, and resilience development—program. Additional institutional support allowed us the opportunity to expand from a base team of dedicated providers in cardiology, hepatology, immunology, and endocrinology by adding a dedicated clinical psychologist, dietician, exercise physiologist, and social worker.

From inception of program in 2011 through September 2021, 626 patient evaluations have been performed. Over the past 5 years, 38% are first-time referrals to FORWARD with 62% returning visits. Overall, approximately 1/3 of patients evaluated have never stepped foot in our center before and have come to CHOP specifically for multidisciplinary evaluation in the FORWARD program.

## The Multidisciplinary Approach: Areas of Focus

A variety of sub-specialty domains of care are relevant to the child and adolescent with a Fontan circulation. In what follows, we share (1) why the discipline is of unique importance in a Fontan circulation? (2) how do we approach evaluation and surveillance in the domain of interest? and (3) pearls of wisdom and unique tips we have learned through our experiences in the FORWARD clinic.

### Cardiovascular

#### Why is This Domain Important?

Cardiovascular assessment and evaluation are the foundation of any Fontan circulation surveillance program. Inadequate ventricular performance, abnormal cardiac rhythm, and elevated venous pressures directly impact all other organ systems and influence overall morbidity and mortality. Optimal cardiovascular status in a Fontan circulation is dependent upon several variables included as part of every multidisciplinary evaluation.

#### How Do We Approach Evaluation?

Cardiovascular assessment starts with an in-depth review of medical and surgical history and complications. Operative notes, past cardiac catheterization data and other diagnostic tests are reviewed. Historical complications are noted including early in life feeding challenges, lymphatic complications, arrhythmias, and any past difficulties with ventricular function, valve leakage, or aortic arch obstruction. A history of thrombus, stroke, seizures, need for ECMO, cardiac arrest, or acute kidney injury is noted. The review occurs prior to the clinic visit, summarized, and discussed with the multidisciplinary team at a pre-clinic group review meeting.

At patient and family contact, a cardiac-focused review of systems is undertaken asking about palpitations, chest pain, dizziness, syncope or near syncope, cyanosis and knowledge of baseline oxygen saturation, exercise intolerance, and any history of swelling or cough with cast expectoration. Medications are reviewed.

At physical examination, vital signs are obtained with emphasis on heart rate, four extremity blood pressure, and oxygen saturation. Note is taken of any dysmorphic features, cardiac auscultation, hepatosplenomegaly, edema, varicosities, and distal extremity hair growth.

Cardiovascular testing includes a 12-lead electrocardiogram at each encounter and a Holter monitor once every 1–2 years, or more frequently if indicated. Echocardiography is performed with focus on systemic venous and pulmonary artery pathways, presence of fenestration and mean gradient across, ventricular and valvar function, and aortic arch. Cardiac MRI is performed once every 2–3 years in adolescents, as sedation/anesthesia is typically not required, with focus on branch pulmonary artery anatomy and blood flow, ventricular ejection fraction, valvar regurgitation fraction, aortic arch caliber, and aortopulmonary collateral burden. Cardiac CT imaging is reserved for individuals with contraindication for MRI, such as in those with a pacemaker. Cardiopulmonary exercise testing with metabolic assessment is performed (age appropriate, typically > 10 years old) to assess for heart rate variability, symptoms with exercise, ectopy, rhythm, oxygen saturations, maximum oxygen consumption, and anaerobic threshold. Blood laboratory testing is obtained, including brain natriuretic peptide. We recommend screening cardiac catheterization for all individuals at 10 years out following Fontan operation, which is combined with a liver biopsy (see below) [5]. This proactive approach provides an opportunity to document important pieces of data such as systemic venous pressures, ventricular end-diastolic pressure, oxygen saturation, and intervene as necessary (i.e., stenting for pulmonary artery stenosis) to optimize patency of pathways.

#### Cardiovascular Tips and Pearls of Wisdom

- No two individuals with a Fontan circulation are exactly alike and each have their own story to tell and trajectory to convey. Which aspects of the story contribute to the variability in outcomes is still unclear.
- At evaluation, detailed characterization is of value but so is the initial “doorway impression.” Well or unwell appearing? Ascites with thin extremities? Poor stature? Obesity?
- Oxygen saturation is important; however, more important is the concept of oxygen delivery. The pursuit of the highest possible oxygen saturation may not be of benefit to every individual with a Fontan circulation. We are not of the view to close fenestrations or pursue the occlusion of decompressing veno-venous collateral vessels if resting oxygen saturations are in the low 90% range, as doing so may increase the impedance for systemic venous return, increase venous congestion or raise central venous pressure. *The optimal Fontan circulation is one that achieves balance and compromises between hosts of potentially competing physiological processes*. An ideal individual may very well be one with an open fenestration, an oxygen saturation of 90–92%, with a compensatory slightly increased hemoglobin in the 14–16 gm/dL range. As such, the needle is thread in maintaining adequate downstream ventricular filling, with reasonable cardiac output to provide for adequate oxygen delivery at the lowest possible price of venous congestion.

### Liver

#### Why is This Domain Important?

**Fig. 1:**
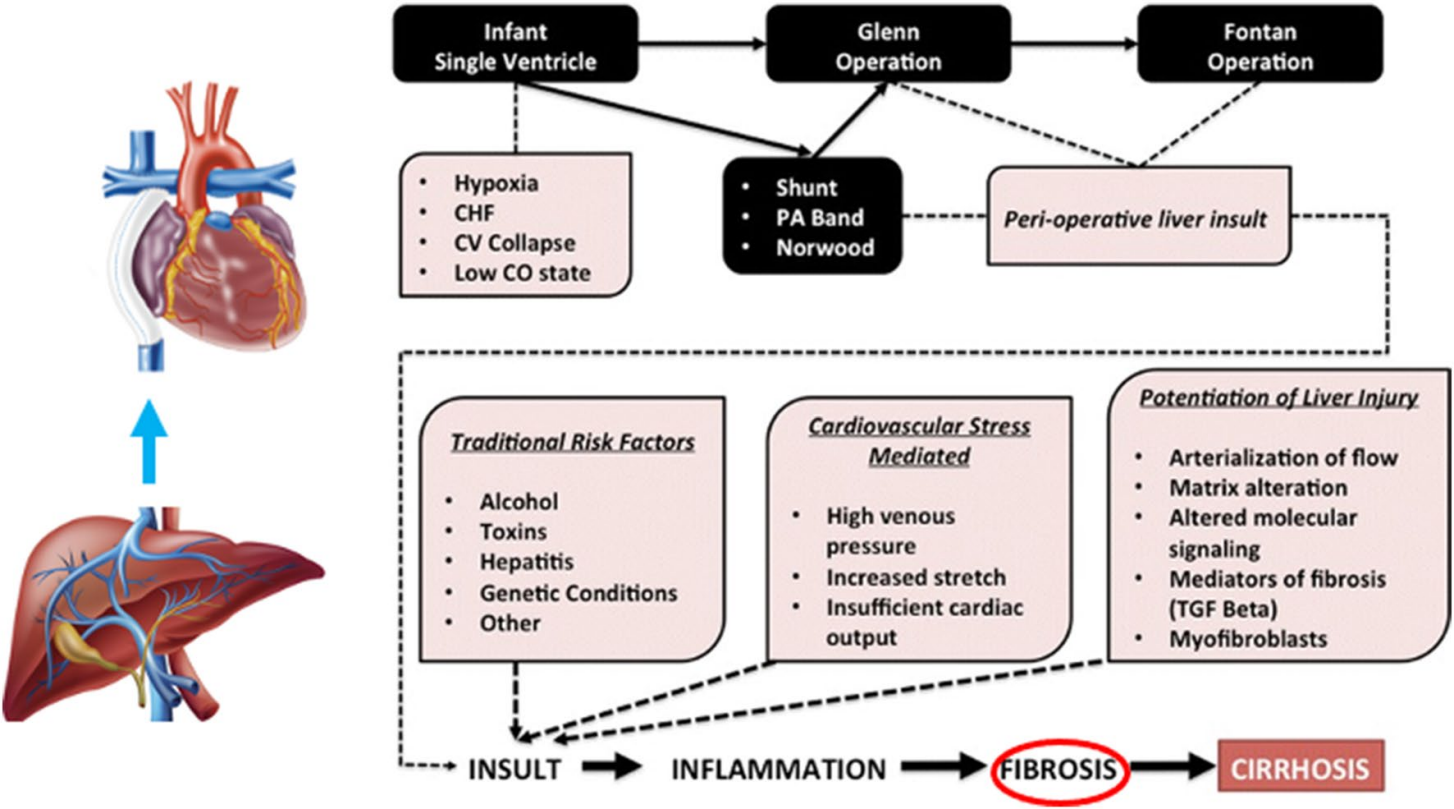
The heart–liver interaction in the patient with single ventricle and potential factors influencing the development of “Fontan-associated liver disease”

It is now widely known that the liver is universally affected in individuals with a Fontan circulation [6]. Inherent differences in hepatic substrate in association with single-ventricle congenital heart disease, injury related to alterations in perfusion along the path of surgical treatment, cyanosis, and congestive hepatopathy as a consequence of chronic venous hypertension due to the Fontan circulatory state may all contribute to what is now referred to as “Fontan associated liver disease (FALD) [7]” (Fig. 1). Hepatic fibrosis is noted on histological examination of liver biopsy and may be progressive with passage of time [8]. The condition poses significant risks with respect to potential compromise of liver function, development of benign liver lesions, and for the risk of liver cancer, specifically hepatocellular carcinoma [9].

#### How Do We Approach Evaluation?

Screening blood work is performed to include liver injury assessments (ALT, AST), gamma gultamyl transferase (GGT), and liver function assessments (bilirubin, albumin, and international normalized ratio (INR)) as well as platelet count as a measure of splenic consumption, related to hepatic congestion (platelet count unhelpful in cases of asplenia). Screening imaging in our practice includes ultrasound, Doppler flow assessments, and elastography as a measure of hepatic stiffness. MRI can also be used for structural imaging and elastography. Blood fibrosis panels used in a variety of other liver conditions are of limited utility, as most are designed for inflammatory and not congestive injury [10]. Ultrasound evaluation often reveals heterogeneous liver echotexture and the findings of benign focal nodular hyperplasia [11]. Blood assessment of alpha feto-protein can be used as a marker for hepatocellular carcinoma and is drawn when imaging reveals new or suspicious hepatic structure and as part of surveillance annually in later adolescence and adulthood. For nodules that appear suspicious on ultrasound imaging, we recommend further imaging with MRI and gadolinium-based contrast (Eovist, Bayer Radiology, Whippany, NJ) to help discriminate between benign nodules and cancer [12].

**Fig. 2:**
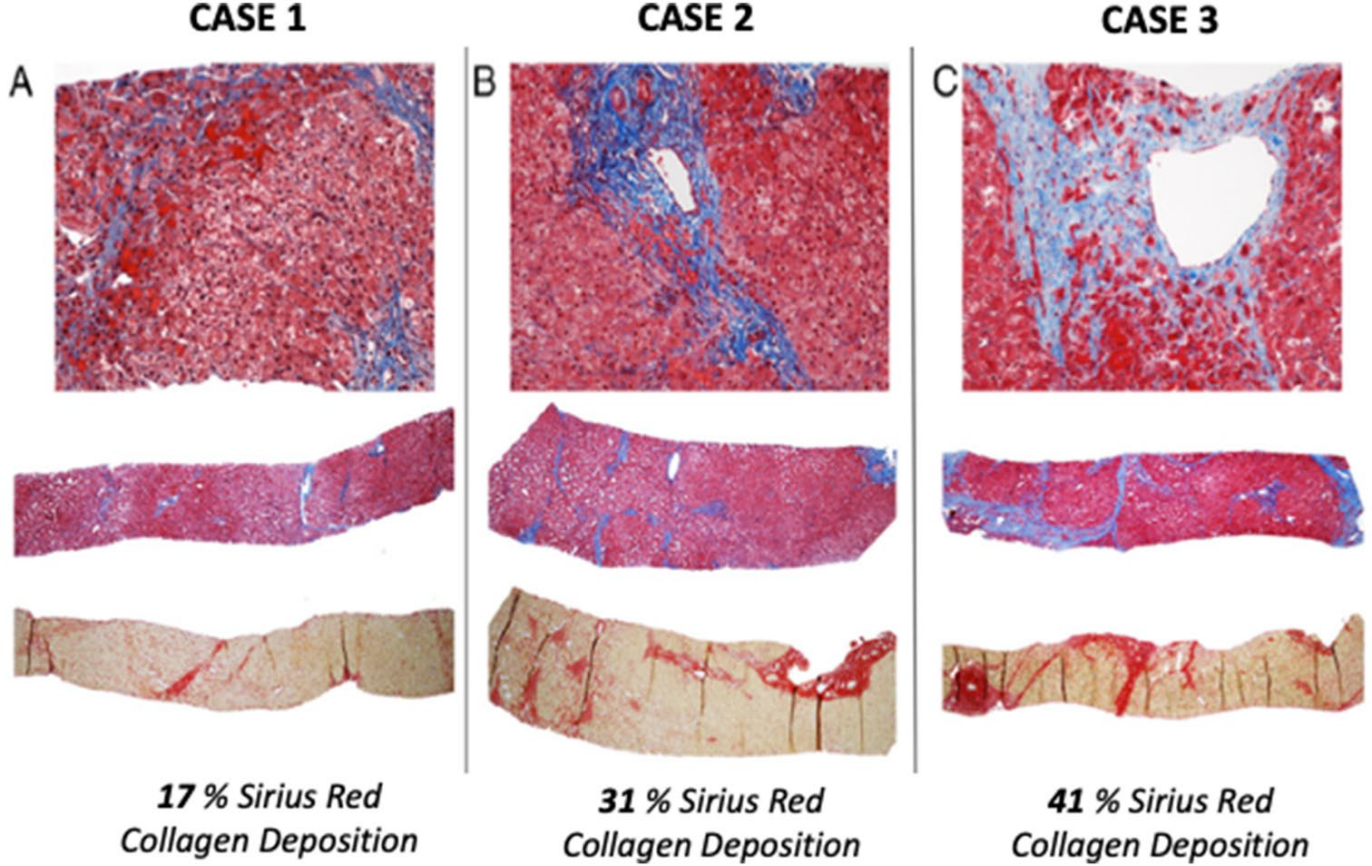
Liver biopsy samples with Sirius red staining for quantification of percent collagen deposition. Utilizing this technique allows for quantification of a global burden of fibrosis per area of tissue analyzed. Case 1 with a minimal amount of fibrosis, and cases 2 and 3 with more significant amounts of fibrosis

Our center is unique in that we recommend a screening cardiac catheterization and liver biopsy in all individuals at approximately 10–12 years out from Fontan operation [5]. While controversial, we believe that a proactive approach for individuals as they enter their adolescent years provides a wealth of important information concerning hemodynamics and liver status. Correlating contemporary liver blood test values and elastography measures to fibrotic histopathology on liver biopsy provides a context for non-invasive measures that can then give meaning to any changes that may be seen on future serial non-invasive evaluations. Liver biopsy information within the context of hemodynamics provides an opportunity to optimize cardiovascular conditions through medical management (e.g., use of pulmonary vasodilators, or aldosterone inhibition). Of note, due to the uniqueness of the Fontan circulatory physiology, hepatic venous wedge pressures do not correlate with degree of fibrosis [13]. We have also applied a novel method in quantifying overall burden of fibrosis in our histopathology samples by using a Sirius red staining technique and semi-automated quantification [14]. This results in a percentage score that reflects magnitude of fibrosis [8] (Fig. 2). Our sampling technique involves percutaneous needle liver biopsy at completion of cardiac catheterization following reversal of anticoagulation [15]. Patients are admitted overnight for observation, with hemoglobin value obtained post-procedure. Utilizing this strategy, we have safely performed over 200 cardiac catheterization and liver biopsy assessments since the start of our program without any serious adverse events.

#### Liver Tips and Pearls of Wisdom

- Despite fears of many cardiologists, most patients/families appreciate learning about FALD. Patients with a Fontan circulation should be regularly screened by a hepatologist familiar with FALD and the benefits and limitations of current assessments.
- INR Values are typically mildly elevated (< 1.5). ALT and AST values are generally 1.5–2.5 times the upper limit of normal. Important to appreciate that these values are in fact “normal” for those with Fontan circulation. GGT values can be significantly higher than 2 times normal. GGT is a ubiquitous enzyme found on the external surface of cellular membranes; thus, markedly elevated values reflect not only liver impact but also extra-hepatic release as seen in cardiovascular disease and conditions of oxidative stress [16, 17].
- Platelet count values are a sensitive marker of portal hypertension in children without asplenia/polysplenia syndrome.
- Elastography overestimates the magnitude of fibrosis as the values are a composite of both hepatic vascular congestion and fibrosis. This is the reason why we recommend cardiac catheterization and liver biopsy to best understand the contribution of each variable—venous pressure and fibrosis—to the elastography values.
- Although there is currently little data to support demonstrated efficacy of any agents to treat FALD, there are drugs known to have salutary cardiovascular as well as antifibrotic properties that may prove to be useful (e.g., spironolactone)
- We have observed patients with Fontan circulation and protein-losing enteropathy may exhibit lower elastography values than those without protein-losing enteropathy. We speculate this occurs as a consequence of lymphatic flow “decompression” into the gut. Elastography values may, thus, be spuriously low and not reflect true degree of FALD present.
- We often use the magnitude of Sirius Red percent collagen staining as a measure of overall burden of fibrosis in our liver biopsies. For example, an increased level of percent Sirius red collagen staining (30–40%) within the context of elevated systemic venous pressures (> 15 mm Hg) on catheterization may prompt consideration of pulmonary vasodilator therapy. Conversely, a relatively low level of percent Sirius red collagen stating (< 10%) with relatively low systemic venous pressure (< 12 mm Hg) indicates favorable findings.

### Endocrinology

#### Why is This Domain Important?

Endocrinological evaluation involves the assessment of growth, pubertal development, bone, and mineral metabolism all of which are at risk in a Fontan circulation. Normal growth in children is a reflection of a healthy state resulting from an equilibrium of multiple genetic, hormonal, nutritional, metabolic, psychosocial, and environmental factors. This is especially relevant in children with a Fontan circulation who may have numerous risk factors for poor growth including hypoxemia, relatively low cardiac output, poor nutrition, and nitrogen-depleting conditions such as the complication of protein-losing enteropathy (PLE). Studies have shown a higher prevalence of short stature in children and adolescents after Fontan compared to the general population [18–20]. Even though, on average, the height deficits post-Fontan are mild, there are subgroups at higher risk for more severe growth problems and it is in these cases where a timely intervention is mandatory to investigate comorbidities and allow time for catch-up growth. Delayed puberty in both genders has been described in adolescents with Fontan physiology, with late but complete pubertal development [19]. Adolescence is a very important time for the accrual of peak bone mass. Although it is the impression from our clinic that we generally do not see an increased risk of fractures in children with Fontan physiology, deficits in bone mineral density (BMD) have been described [21, 22]. Given the increased likelihood of survival into adulthood, it is important to optimize bone health at a young age. Increasing physical activity is one of the most important factors in improving bone health in those with a Fontan circulation.

#### How Do We Approach Evaluation?

The endocrine evaluation starts with a thorough history and physical exam. Family history will inform us of mid-parental height, a genetic predisposition to constitutional delay in growth and development, autoimmune or gastrointestinal disorders. Growth velocity is the most important parameter which defines if a child is healthy from an endocrine standpoint. Prior measurements from the pediatrician will be needed to determine if there is a pathologic decrease in height percentile, or a late growth spurt due to delayed puberty. Assessment of weight and body mass index BMI will tell us if there are nutritional causes for poor growth, which could be due to gastrointestinal disease or behavioral in nature. Physical exam will reveal dysmorphia which could be related to a genetic syndrome associated with short stature (3.3% of our recent series). Tanner staging is part of the examination in all adolescents.

If the growth pattern is abnormal, the initial growth evaluation includes a bone age X-ray, blood labs including comprehensive metabolic, complete blood count, erythrocyte sedimentation rate, celiac panel, thyroid function tests, and growth factors. In the presence of dysmorphia, a genetic evaluation would be warranted. In the case of short stature with poor weight gain, a nutritional evaluation would be the first step, with close follow-up. Pubertal delay would be further evaluated with gonadotropin and sex steroid levels.

As is the case in the general population, some children with Fontan physiology can have growth hormone (GH) deficiency. These are usually patients with normal or high BMI and normal testing except for low growth factors. In this situation, the diagnosis is confirmed with a growth hormone stimulation test and work-up is completed with a pituitary MRI and assessment of other pituitary hormones.

Regarding bone health, DXA scans are part of routine testing in our clinic, as well as blood lab comprehensive metabolic, 25 OH Vitamin D, and parathyroid hormone levels. If BMD is low for age, we must assure adequate intake of calcium and vitamin D and prescribe supplements in cases of insufficient 25 OH Vitamin D levels, low intake of calcium by dietary history or reflected by secondary hyperparathyroidism (increased parathyroid hormone levels).

Patients with PLE on steroid treatment with budesonide require assessment of their adrenal axis [23]. A first screening test is a morning AM cortisol level, but a definitive test would be a low dose ACTH or CRH stimulation test.

#### Endocrine-Based Tips and Pearls of Wisdom

**Fig. 3:**
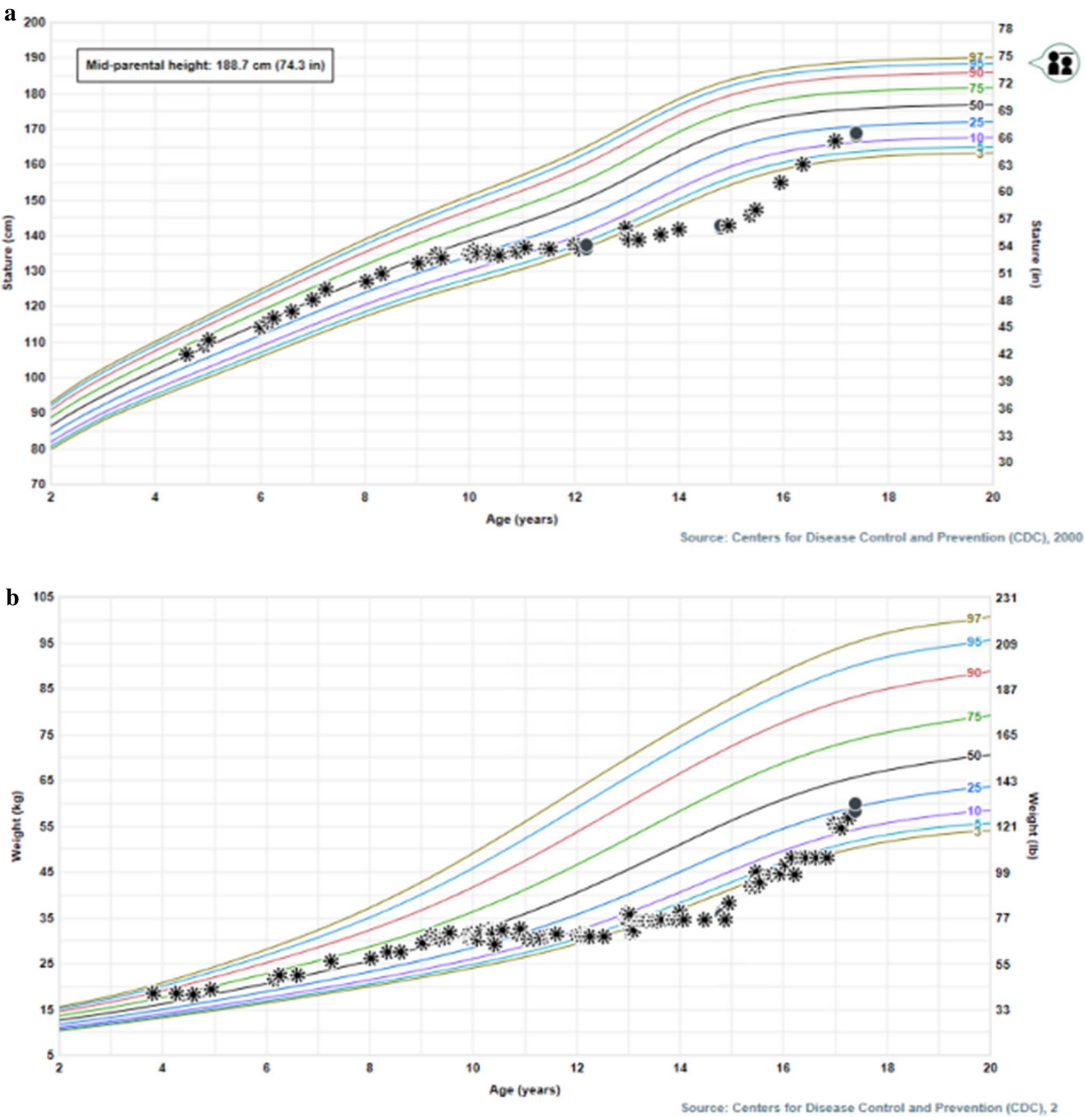
Growth charts of male patient with Fontan physiology, complicated by PLE at age 9y, treated with diuretics and steroids (oral controlled release budesonide) with subsequent growth failure and delayed puberty. Growth charts show catch-up growth with a delayed pubertal growth spurt after resolution of PLE, budesonide wean and improved nutritional intake. **a** Height for age curve, **b** weight for age curve

- Children with Fontan physiology have a higher prevalence of short stature than the general population and this is often due to delayed growth with late puberty, needing no specific treatment but reassurance. As in the general population, when growth velocity is low, a work-up as described above is mandatory. In many cases, poor weight gain is the cause of the delay in growth and nutritional intervention leads to improvement. Children with genetic syndromes and complications such as PLE are the 2 groups in whom height is affected to a larger degree.
- In the cases complicated by PLE, growth improves with improvement of PLE, nutritional support, and cessation of steroid treatment. In Fig. 3, we display the growth curves of a male patient with PLE diagnosed at age 9 years, treated with diuretics and budesonide with subsequent growth failure and delayed puberty. Growth curve shows catch-up growth with a delayed pubertal growth spurt after improvement in PLE, budesonide wean, and improved nutritional intake.
- Many families ask us about growth hormone treatment for short stature. As in the general population, the minority of short children have GH deficiency and require GH treatment. We have prescribed GH in a small number of children diagnosed with GH deficiency. However, we do not recommend GH for Idiopathic Short Stature (ISS) or children with lymphatic complications such as PLE because GH has led to recurrent exacerbations of PLE in the Fontan circulation [24].
- A healthy nutrition and lifestyle and plenty of physical activity are the best advice we give to improve both growth and bone health in our children with Fontan circulation. Families of children with Fontan often think they should not be as physically active as other children and we always explain the beneficial effect of weight-bearing exercise such as walking and jogging on bone health and health in general.

### Immunology

#### Why is This Domain Important?

Immunologic complications of Fontan physiology can include hypogammaglobulinemia and lymphopenia, particularly of naïve T-cells. In patients with PLE, secondary intestinal lymphangectasia leads to loss of serum proteins and lymphocytes [25]. While production of lymphocytes and immunoglobulin is normal, in those with PLE, loss into the bowel surpasses the rate of production. Lymphopenia without hypogammaglobulinemia has also been described in those without PLE [26]. We recommend immunologic evaluation for all individuals with a Fontan circulation in our FORWARD clinic.

#### How Do We Approach Evaluation?

Immunoglobulin and lymphocyte assessments are performed. The hypogammaglobulinemia seen in Fontan circulation is generally considered a quantitative defect, while qualitative antibody function (measured by assessment of vaccine titers) is typically normal. Our group and others [26, 27] have described that despite median IgG levels of 100–200 mg/dL (normal median > 500 mg/dL), most patients do not have significant sinopulmonary or bacterial infections, typically seen in other patients with hypogammaglobulinemia.

Based on this, we typically screen all patients, regardless of PLE status, with assessment of clinical infection history and measurement of serum IgG level. In those with lower IgG than normal, we will follow up with assessment of tetanus and pneumococcal titers as well as quantitative IgA and IgM levels. For patients with hypogammaglobulinemia and recurring infections, subcutaneous (rather than intravenous) immunoglobulin infusions may be beneficial to reduce infection while avoiding giving large fluid volumes. Important to note that we do not treat hypogammaglobulinemia unless there is an associated problem with recurrent infections. If infection history is present, we typically initiate use with weight-based dosing (150 mg/kg/week) and will titrate to a dose that limits infection. Achievement of typical IgG levels of 800–1000 mg/dL for patients on replacement therapy in the context of PLE is often not feasible due to ongoing losses.

Selective T-cell lymphopenia is the most common lymphopenia we have observed, typically leading to preferential loss of naïve (CD4/CD45RA) T-cells. Additionally, in patients with more severe PLE, B-cell lymphopenia has also occasionally been seen which improves as the PLE is controlled medically. Despite remarkably low overall T-cell counts (median CD3 672 cells/uL, normal > 1000 cells/uL), the patients typically do not suffer opportunistic infections, such as thrush, mycobacterial infections, or pneumocystis pneumonia. We have also described that, for patients without the complication of PLE, T-cell lymphopenia is commonly seen in those who are more than 10 years post-procedure. Since these patients also likely had at least partial resection of the thymus, this may also play a role in the later onset T-cell lymphopenia of non-PLE patients, but this has not yet been fully explored. Interestingly, we observe that 25% of individuals with Fontan circulation experience significant viral cutaneous infections (warts and/or molluscum lesions. Cutaneous viral infections do not correlate with overall lymphocyte, T-cell or natural killer cell (NK) counts. Further studies are ongoing to better understand the cell-based physiology of this phenomenon.

Based on this, we typically screen all patients, regardless of PLE status, with measurement of absolute lymphocyte count (ALC). In those with ALC less than 1000 cells/uL flow cytometry-based enumeration of T-cells (CD3, CD4, CD8), B-cells (CD19), NK-cells (CD3-/CD56 and/or CD16) is performed with assessment of naïve (CD4/CD45RA) and memory (CD4/CD45RO) T-cells as well. Antibiotic prophylaxis directed at potential opportunistic infections is not generally indicated so long as the infectious history is reassuring.

Due to the higher-than-expected frequency of significant challenges with warts, we do assess this history and perform careful physical exam assessment of hands and feet. If warts are present, we typically recommend a brief trial of over-the-counter remedies with a low threshold for dermatology evaluation and treatment if there is not a timely clinical response.

#### Immunology-Based Tips and Pearls of Wisdom

**Fig. 4:**
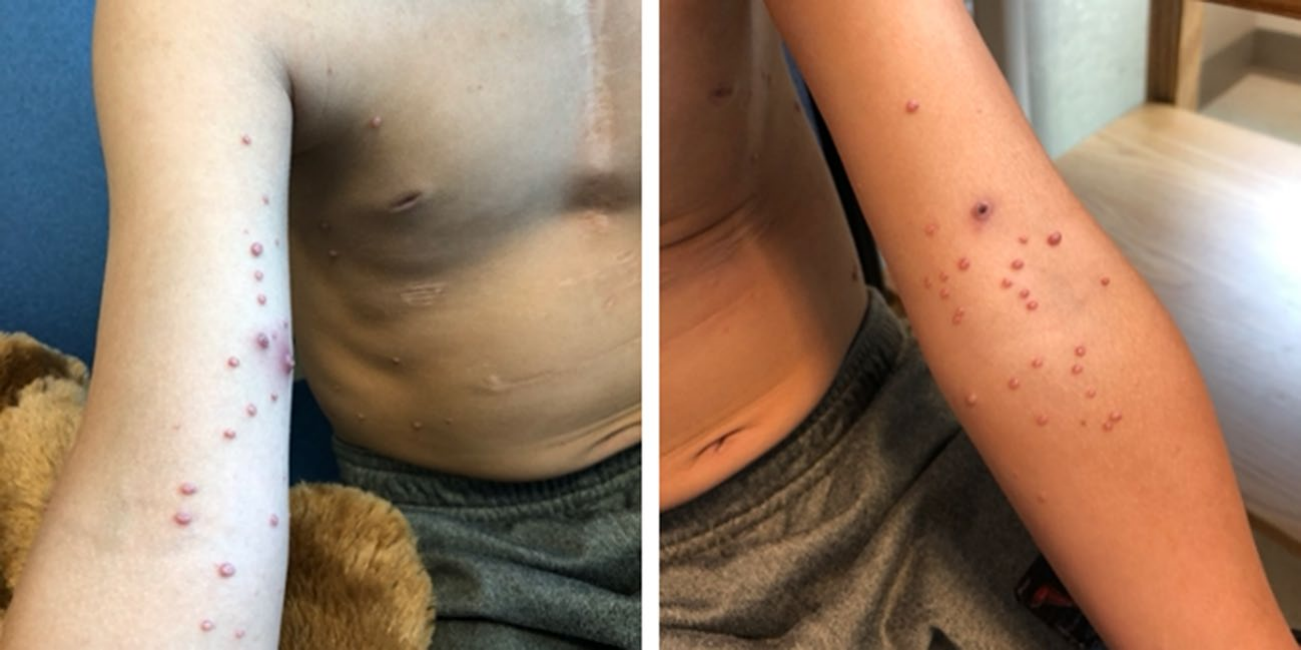
Eight year old with Fontan circulation and extensive molluscum contagiosum on both arms

- We do recommend administration of all routine immunizations, including live vaccines such as MMR and Varicella to all post-Fontan patients, regardless of their T-cell counts. Annual influenza vaccination, as well as COVID-19 mRNA-based vaccine are also recommended following CDC guidance.
- In some cases, viral warts can be severe, with excoriation leading to the potential for secondary infection (Fig. 4).

### Behavioral Health and Psychology

#### Why is This Domain Important?

Children with congenital heart disease are at increased risk for having emotional and behavioral challenges compared to typically developing children. Specifically, they are four to five times more likely to have an anxiety or depressive disorder and at least twice as likely to have attention deficit hyperactivity disorder (ADHD) [28]. Youth with Fontan circulation are at even greater risk for these problems, with one estimate of lifetime prevalence of psychiatric disorders of 65%. This cohort is distinct in this regard, as the likelihood of mental health concerns is greater than what is observed in other chronic illness populations [29]. Furthermore, these children are at increased risk for neurodevelopmental problems, including learning disabilities, intellectual disability, and executive function deficits, even when compared to children with other forms of CHD [30]. Mental health concerns are a major component of unwellness among individuals with Fontan circulation; however, there is little understanding about what specific medical, psychological, and sociodemographic variables are most predictive of these problems.

Over the last few decades, there has been increasing interest in the integration of mental health professionals into healthcare teams, including pediatric sub-specialty care. Inclusion of psychologists in medical teams has been shown to have high levels of satisfaction with care for both patients/families and medical providers [31]. In addition, there is also evidence to suggest that integration of psychologists into medical teams improves patient mental health and medical outcomes as well as being cost saving for healthcare systems [32, 33]. Given the mental health challenges, many youth with Fontan circulation face, incorporating professionals, including psychologists and social workers, to help assess then address these needs is essential and clearly has the potential to benefit patient outcomes.

#### How Do We Approach Evaluation?

A dedicated psychologist provides mental health evaluations for all patients that are seen in the FORWARD clinic. Evaluations include sessions with patients and their parents. The domains assessed include mental health history, current psychological concerns, educational history, medical adherence, patient knowledge about their medical history, and coping with medical procedures. Evaluation also includes parent and child completing standardized psychological questionnaires, a broad-screening measure of various emotional/behavioral challenges (Behavioral Assessment Scale for Children, Third Edition [BASC-3]) as well as health-related quality of life measures (including Pediatric Cardiac Quality of Life Inventory [PCQLI]) [34]. These questionnaires are sent to families via email in advance and completed prior to on-site appointment.

At completion of evaluation, the psychologist gives recommendations to patients and their families. These commonly include suggestions for families to support the patient in having greater knowledge about their medical history and strategies for gradually increasing the patient’s responsibility for self-management of their health in a developmentally appropriate manner. If the patient has learning problems or other neurodevelopmental issues that are not being sufficiently addressed, families are often encouraged to ask for a psychoeducational evaluation through the child’s school. If these problems persist despite school support or if there are more complex neurocognitive concerns, patients are referred for specialized neuropsychological testing. For minor emotional/behavioral challenges, the psychologist will provide psychoeducation on cognitive-behavioral strategies to address these issues which families are encouraged to implement themselves. For more significant emotional/behavioral problems, families are referred to mental health treatment. Depending on the needs of the patient, they may be referred to find local mental health support through a therapist in the community for standard emotional/behavioral concerns such as depression and anxiety. For issues related to medical adherence or significant emotional/behavioral challenges that are directly related to the medical condition, families are referred to more specialized treatment with a pediatric psychologist. If a referral for psychotropic medication management (e.g., SSRIs, psychostimulants) is warranted, the psychologist will often first discuss with the medical team regarding safety of these medications and any additional monitoring that would need to be done during a medication trial, and then this information is communicated back to the family. Families are typically encouraged first to follow up with the patient’s pediatrician for evaluation and then are referred to a child psychiatrist or developmental pediatrician if needed. The psychologist also often helps coordinate care and facilitate communication related to treatment of mental health issues among the sub-specialty medical providers, pediatricians, and community mental health providers.

#### Behavioral Health and Psychology Based Tips and Pearls of Wisdom

- Many of the patients that come through the program have never met with a psychologist before. Some families have been traumatized by their medical and surgical experiences, with both patient and parental anxiety and stress levels commonly elevated. The vast majority of patients/families are agreeable to meet with the psychologist, and many express appreciation for the incorporation of mental health as part of the comprehensive evaluation they are receiving in clinic. If patients or families express doubt or uncertainty about whether they need to meet with psychology, they are provided psychoeducation about the role of psychology in this context. That is, in addition to a focus on mental health, which many patients expect, the psychologist also addresses issues related to the child’s medical condition and neurodevelopment.
- A key element of our program is that it provides truly integrated care for patients. While there are many models for how psychologists can be integrated into healthcare teams, fully integrated care involves ongoing discussion and collaboration with sub-specialty physicians and other clinicians to create a unified treatment plan that considers all aspects of the patient’s healthcare needs [35]. Mental health goals and recommendations are not pursued simply in parallel with physical health treatment, and they are integrated into the conceptualization of the patient’s needs through collaborative, face-to-face, discussions with the other medical providers.
- Due to the complex needs many patients express, the psychologist often engages in care coordination and consultation outside of the context of clinic visits. Some families need additional guidance about getting connected with local mental health resources as well as education regarding evidence-based treatment strategies for specific problems we often encounter, such as selective mutism, intellectual disability, and autism.
- Psychological assessments are just one of many forms of evaluation to take place at FORWARD clinic visit. Since onset of the COVID-19 pandemic, there has been a need for greater flexibility in terms of scheduling. As such, the program adopted a model in which many families can engage in a telehealth evaluation with the psychologist at a scheduled time prior to the on-site clinic visit.

### Nutritional Health

#### Why is This Domain Important?

Optimizing nutritional status is essential for health and well-being for individuals with Fontan circulation. The most common nutritional concern is vitamin and mineral deficiencies related to bone health, specifically low vitamin D intake, low serum vitamin D, and inadequate dietary calcium. Bone health is of concern in this population given the use of medications that are detrimental to bone development such as diuretics, anticoagulants such as warfarin, and steroids as well as the overall low activity level that is seen among many patients. Another common nutrition concern is both underweight/malnutrition and obesity. Obesity is a risk factor for poor performance and negative outcomes in the Fontan circulation [36]. Among our underweight/malnourished patients, the concern is primarily related to linear growth deficits and a lack of nutritional reserve in the case of frequent illnesses and/or hospitalizations. At last, there is a small subset of patients with lymphatic leaks resulting in PLE and chronic nitrogen wasting who require careful nutritional attention and care to maintain muscle mass.

In addition to our individualized nutrition recommendations, we also encourage our patients to consume a diet rich in fruits, vegetables, whole grains, low-fat dairy, and lean proteins for long-term cardiovascular health.

#### How Do We Approach Evaluation?

Biometrics of height, weight, and BMI are recorded to assess growth and degree of underweight, overweight, or obesity. Diet is examined for nutritional adequacy particularly focusing on vitamin D and calcium which tend to be nutrients of concern in this population given their role in bone health. In more concerning patients, such as those with potential malnutrition and/or PLE or where there is parental concern, we request a 3-day food record and complete a thorough computerized diet analysis.

#### Nutrition and Dietary Tips and Pearls of Wisdom

- There is no special “Fontan diet.” For the majority of our patients, we recommend a diet to optimize cardiovascular health that includes fruits, vegetables, whole grains, low-fat dairy, and lean protein sources in addition to limiting food sources of added sugars, sodium, and saturated/trans fats and including food sources of fiber, unsaturated fat, and omega 3 fatty acids. This dietary pattern is no different than what is recommended in the USDA’s dietary guidelines.
- For cyanotic individuals, maintaining sufficient iron stores to optimize hemoglobin production is important. Iron supplementation is often recommended if hemoglobin levels appear relatively low in proportion to oxygen saturation, or if laboratory data support low iron stores [37].
- For patients with PLE, medical therapy is of primary importance, and diet alone does not treat the condition [38]. Nevertheless, in some cases we also recommend a very low-fat (< 10% of calories, 20–30 g fat), high protein (> 2 g/kg) diet with the addition of MCT oil for extra calories and essential fatty acid supplementation. It is important to note that this low-fat diet should only be used during acute PLE flares and not long term as we have seen patients struggle to gain weight when following a low-fat diet for a prolonged period of time.

### Exercise and Activity

#### Why is This Domain Important?

Patients with Fontan circulation experience decreased exercise performance with associated increased morbidity and mortality. By the early second decade of life, maximal oxygen consumption (VO2) is typically significantly lower compared to their healthy peers (about 65% predicted) [39] with progressive declines over time in peak V02, percent predicted VO2, and percent predicted peak work rate [40]. There is a significant negative correlation between peak VO2 and age. These declines are worsened by obesity which is associated with decreased physical activity in children with Fontan physiology [36].

It is well known that regular physical activity is an important tool in preventing obesity and slowing the decline in exercise capacity, as well as supporting mental health and overall wellness. Establishing a regular exercise routine early in life is important to maintaining levels of exercise performance throughout adolescence and adulthood, as well as aiding in obesity prevention. Exercise counseling is especially important in the population with Fontan circulation to ensure that these patients are aware of the impact of physical activity on their long-term health. Aside from the well-known benefits of exercise in the general population, it is important to educate on the specific types of exercise that are particularly helpful for those with Fontan circulation Weight bearing exercise for maintaining bone density is important. Greater leg lean mass has been shown to be associated with higher peak VO2. Therefore, exercise focusing on lower extremity strength training is of particular importance [41, 42].

#### How Do We Approach Evaluation?

Cardiopulmonary exercise testing is an important tool in assessing baseline exercise performance, as well as monitoring changes over time as an aid in clinical decision making. Measures of oxygen consumption, physical working capacity, electrocardiography, blood pressure response, oxygen saturations, and pulmonary function are assessed with utilizing either the cycle ergometer or treadmill. Six-minute walk testing can be performed in patients who are either too young or have development physical limitations that make performance of maximal exercise testing inappropriate. Distance can be tracked over time. Heart rate response, blood pressure response, and oxygen saturations with 6-min walk are also assessed.

Following exercise testing, exercise counseling is provided. A dedicated FORWARD program exercise physiologist meets with the patient and family to counsel on safe and effective ways to improve exercise capacity. Special attention is made on weight-bearing exercise and lower extremity strength training in addition to regular aerobic exercise. Patients are led through a sample resistance training program they can do at home. The exercise physiologist and the family work together to develop a regular exercise routine with the resources and exercise equipment available to the family. An emphasis is made on the importance of implementing a regular exercise regimen in patients who are sedentary. For those actively involved in recreational or competitive sports, an emphasis is made on preparing to transition to a life-long exercise routine, when they are no longer involved with team sports.

#### Exercise and Activity Related Tips and Pearls of Wisdom

- One of the most common misconceptions heard from patients and families is “I’m not allowed to play sports” or “I’m not allowed to lift weights.” The exercise training session focuses on advising patients and their families on the benefits of safe and appropriate exercise as opposed to what activities to avoid (highly isometric exercises). Families are often very surprised and excited to learn that their child can actually exercise more than previously thought and the patients are often very excited to learn they can use that treadmill in the basement or lift light weights!
- Exercise is medicine for the Fontan circulation [43]. Determining the ideal type and proper “dosage” of exercise that leads to the most valuable personalized result for any one individual, still requires further exploration. Nevertheless, general exercise and activity are to be strongly encouraged and is a focus of much conversation in our FORWARD clinic encounters.

### Additional Collaborative Institutional Programs

At our institution, neurodevelopmental evaluation in infancy and childhood is provided by a dedicated team of pediatric cardiologists, neurologists, and developmental specialist providers focused on congenital heart defects. The FORWARD team takes advantage of the benefit of a dedicated pediatric neurologist with expertise in the realm of congenital heart disease for complications such as non-cardiac related dizziness, stroke, and migraine headache. Similarly, nephrology services are solicited on an as needed basis from a dedicated pediatric kidney specialist. Through this collaboration, we reported on the very first ever successful living-related donor kidney transplant in a Fontan circulation [44]. For patients with PLE and plastic bronchitis, we have the benefit of the pioneering work of partners in our CHOP Lymphatics Program [45, 46]. Novel techniques for diagnostic lymphatic evaluation as well as life-saving interventional procedures are available to patients in need. Close collaboration with heart failure and transplantation services exists. In a two-way manner, patients referred to the heart failure and transplant program are often sent to FORWARD for detailed characterization of organ systems, as well as individuals seen in FORWARD may be referred to the heart failure transplant team for evaluation. For the adolescent, risky behaviors and birth control are addressed at FORWARD clinic encounters in an age-appropriate manner. Transition to the adult congenital heart disease program commences at various adolescent and young adult ages, with an eye towards creating measures to sustain life-long future health and wellness in all of the sub-specialty domains unique to the Fontan circulation.

### What is It Like to Come to the Fontan FORWARD Multidisciplinary Clinic?

#### The Mechanics of an Evaluation

An evaluation in the FORWARD clinic involves two phases, the testing/evaluation phase and the provider encounter phase. Table 1 is a summary of the various assessments and tests offered to individuals coming to a FORWARD clinic visit. Testing takes up one full day of time and is typically completed prior to the multidisciplinary clinic visit. For local patients, this can potentially be broken up and scheduled at convenience within a few days or weeks of the visit. For out of area patients, the assessments/testing are typically completed in the 1–2 days prior to the visit. Testing is coordinated by the program nurse practitioner who meets with the patient and family, obtains a detailed review of systems history and shepherds them through the evaluation phase.

**Table 1:** Evaluations offered at the FORWARD clinic multidisciplinary evaluation. Multidisciplinary evaluations are suggested following Fontan operation, every 3 years in childhood and every 2 years in adolescence

Clinical domain	Testing	Frequency of surveillance?
Blood Laboratories	Complete blood count with differential	At each visit
	Comprehensive metabolic panel (includes electrolytes, BUN and creatinine, calcium, liver function tests, total protein, albumin)	At each visit
	Cystatin C	At each visit
	Gamma-glutamyl transferase	At each visit
	Parathyroid hormone	At each visit
	25 OH Vitamin D	At each visit
	Brain natriuretic peptide	At each visit
	IgG Immunoglobulin	At each visit
	Alpha feto-protein	When imaging reveals suspicious hepatic structure and routinely in adolescence
	Stool alpha-1-antitrypsin level	When there is low serum albumin levels; suspicion of, or monitoring for PLE
Cardiovascular	Electrocardiogram	At each visit
	Echocardiogram	At each visit
	Holter monitoring	At each visit
	Exercise stress test	At each visit. Without metabolic assessment starting at approximately age 7, with metabolic assessment approximately age > 9 years
	Six-minute walk test	At each visit for young patients and for those unable to do full exercise test with metabolic assessment
	Cardiac MRI	As indicated based on clinical needs, and routinely performed every 2-–3 years when achievable without sedation (typically > age 11 years)
	CT angiography	As indicated based on clinical needs and when MRI not feasible
Liver	Abdominal ultrasound imaging with Doppler	At each visit
	Ultrasound elastography	At each visit (ultrasound is our standard choice)
	MRI elastography (no contrast)	At each visit, in those who have had prior MRI elastography for consistency in serial evaluation
	MRI imaging with gadolinium-based contrast	In the presence of suspicious lesions on ultrasound
Immunology	Flow cytometry-based enumeration of T-, B-, and NK-cells	In those with absolute lymphocyte counts < 1000 cells/microliter
Endocrinology	Dual X-ray Absorptiometry Scan (DXA)	At each visit
	8 AM Cortisol level	Patients with PLE on steroid therapy
	Bone age X-ray	Only as clinically indicated, growth concerns
	Thyroid studies (Thyroid stimulating hormone, free T4)	Only as clinically indicated, growth concerns and in those with PLE
	Insulin-like growth factor 1 and Insulin-like growth factor binding protein 3	Only as clinically indicated, growth concerns
Nutrition	24-h dietary recall	At each visit
	Computerized dietary analysis of 3-day food record	When clinically indicated, growth concerns
Behavioral Health/Psychology	Behavioral assessment scale for children [BASC-3], Parent, and Self-Report	At each visit
	Pediatric Cardiac Quality of Life Inventory [PCQLI]), Parent and Self-Report	At each visit
	PROMIS Global Health/Quality of Life	At each visit
	Brief Pediatric Inventory for Parents (measure of parental distress related to child’s medical condition)	At each visit

Historical information and testing data results are summarized in a document and shared with the providers at “pre-clinic” case review conference held either the day before or morning of the multidisciplinary clinic. All providers are present and offer comments and input. Some sub-specialists such as psychologist, dietician, exercise physiologist, or social worker will have already met the patient and family and may have comments and insights to share. The patient and family are placed in a room in our Cardiology outpatient clinic facility and each of 4 medical sub-specialty providers (cardiology, hepatology, endocrinology, immunology) serially engage. Thus, the patient and family remain in the room while the providers rotate through and have their encounters. In this manner, we can accommodate four patients in the multidisciplinary clinic per half day of FORWARD clinic. Following completion of patients being seen by all providers, a brief wrap-up review meeting is conducted summarizing our findings and recommendations for next steps. Shortly thereafter, a cumulative summary document of the FORWARD evaluation is collated by the program nurse practitioner and provided to patient and referring providers.

As far as timing for evaluation, we currently recommend multidisciplinary evaluation in FORWARD clinic once every 3 years following Fontan operation, up until adolescence where once every 2 years is suggested.

## Next Steps and the Future?

**Fig. 5:**
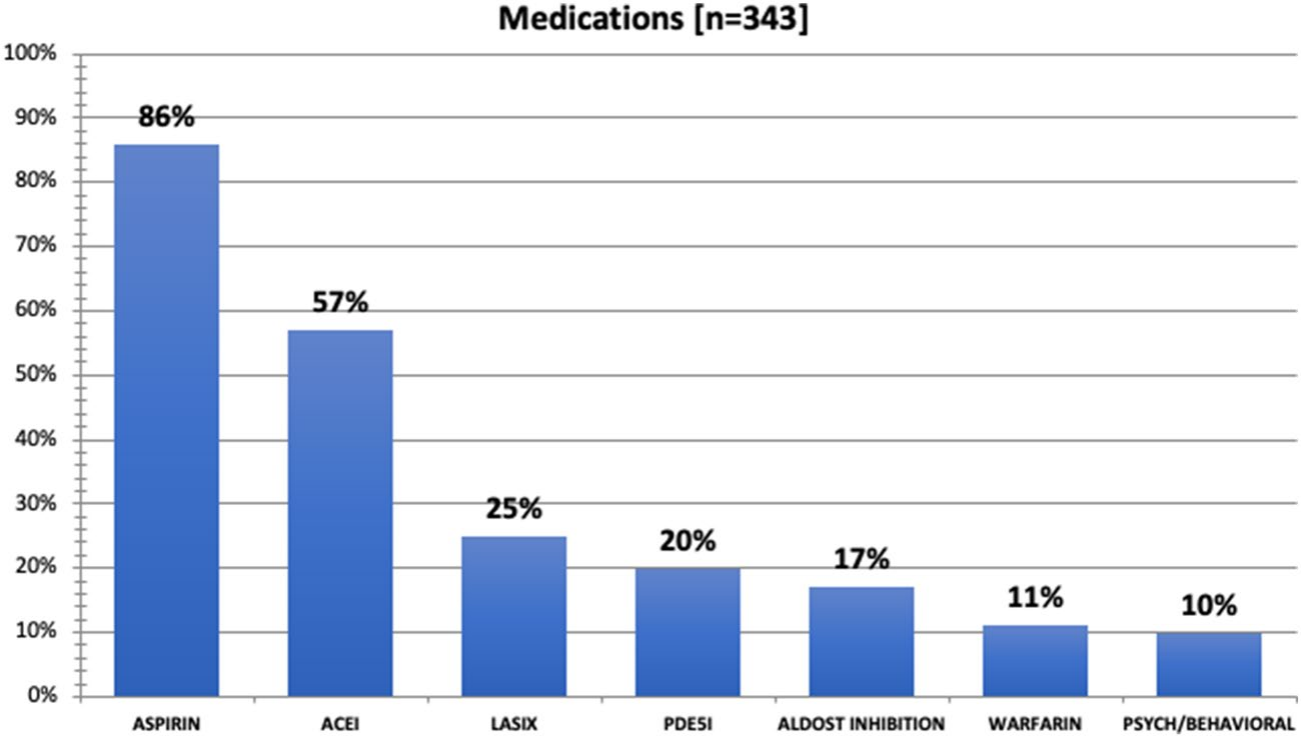
The medication distribution in a sample of 343 patients seen in FORWARD clinic. ACEI, angiotensin converting enzyme inhibition; *ALDOST* aldosterone; *PDE5I* phosphodiesterase—5—enzyme inhibition. Note 10% of our population in this sample are on psychiatric or behavior modifying medications

**Fig. 6:**
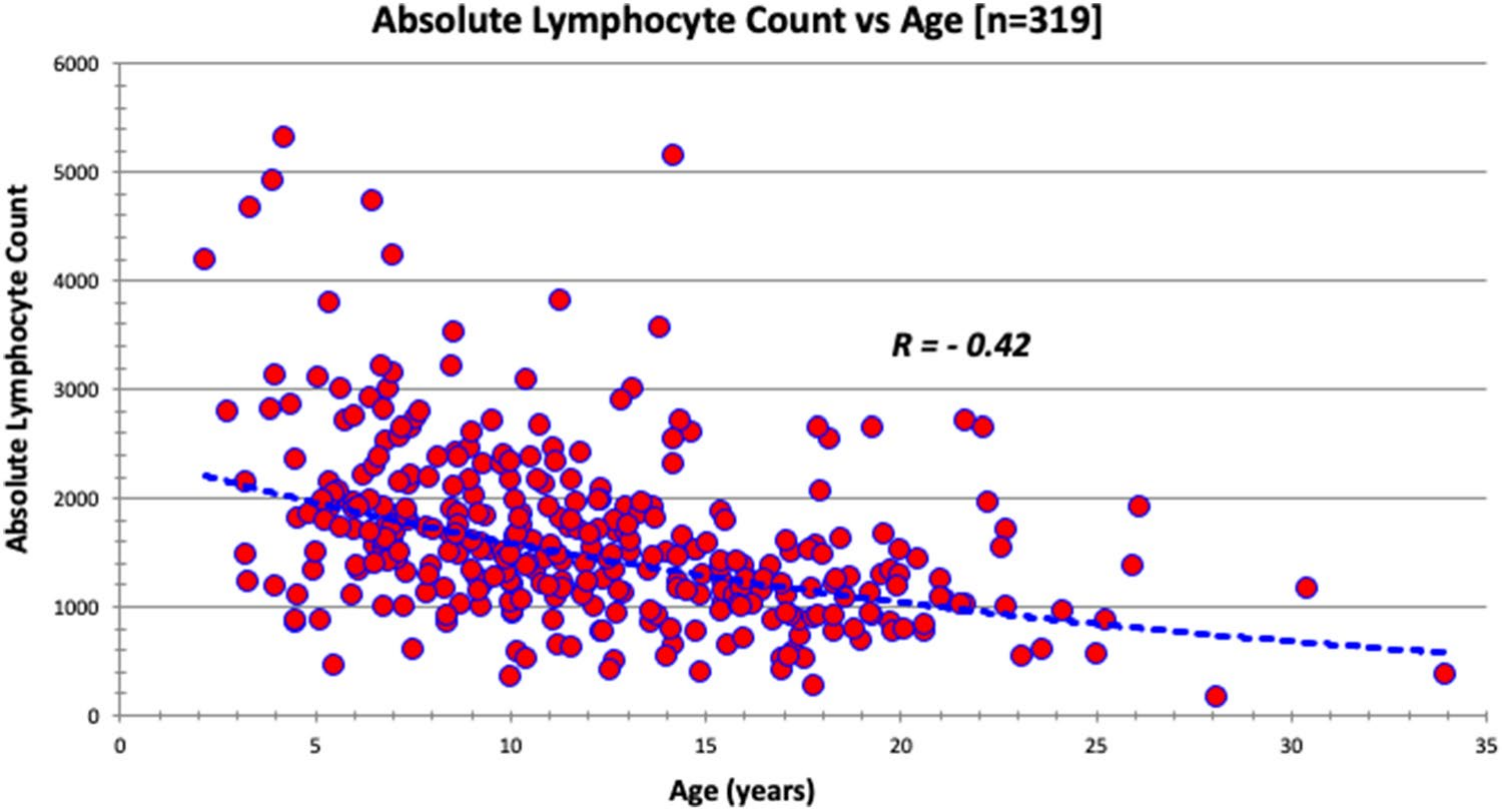
The absolute lymphocyte count (cells per microliter) distribution based on age at evaluation in a sample of 319 patients evaluated in FORWARD clinic. Normal value for lower limit of absolute lymphocyte count in our laboratory is 1160 cells/microliter. The curve is significant at *p* value < 0.01

**Fig. 7:**
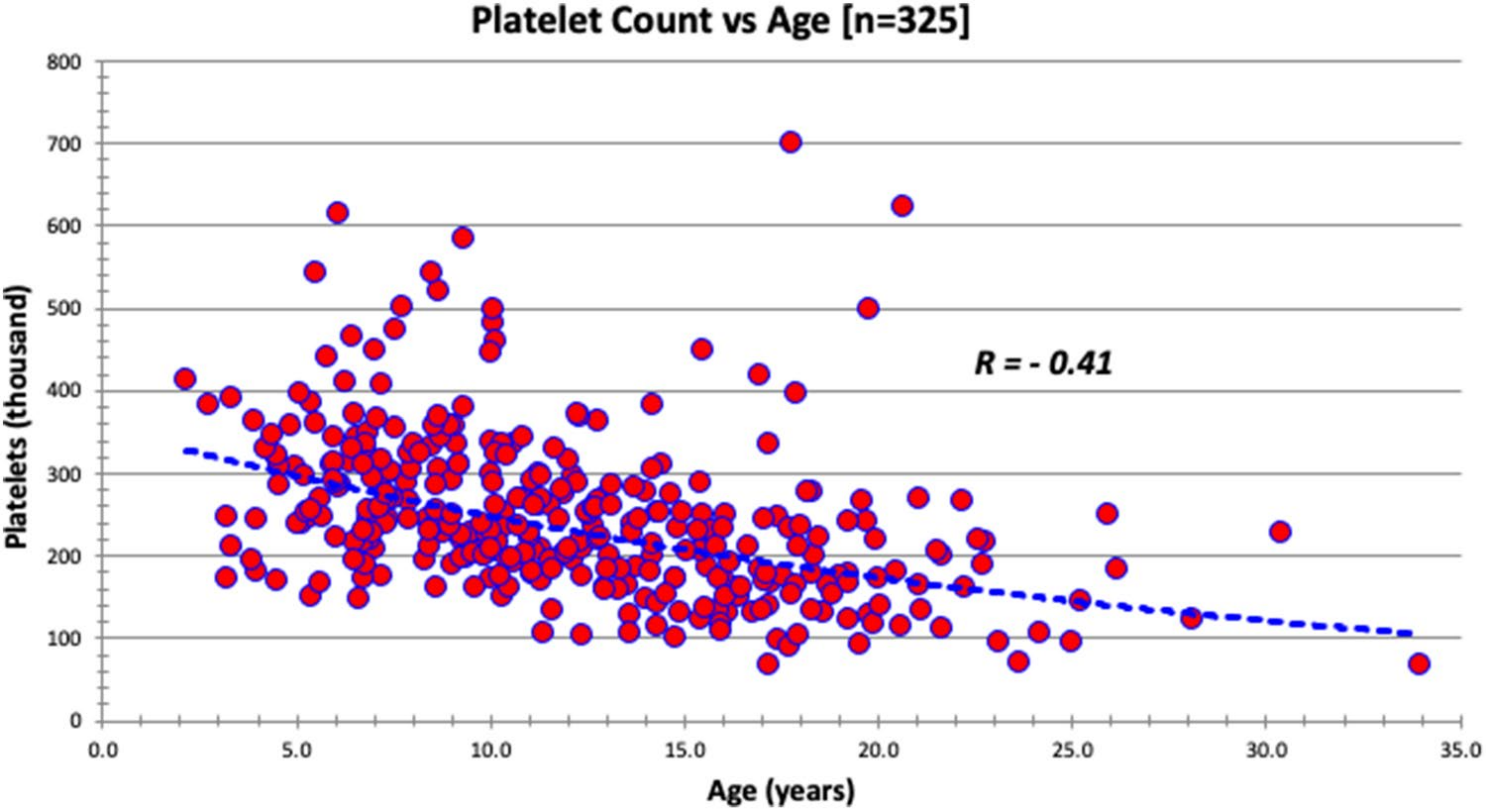
The platelet count (10*^3^ per microliter) distribution based on age at evaluation in a sample of 325 patients evaluated in FORWARD clinic. The curve is significant at *p* value < 0.01

**Fig. 8:**
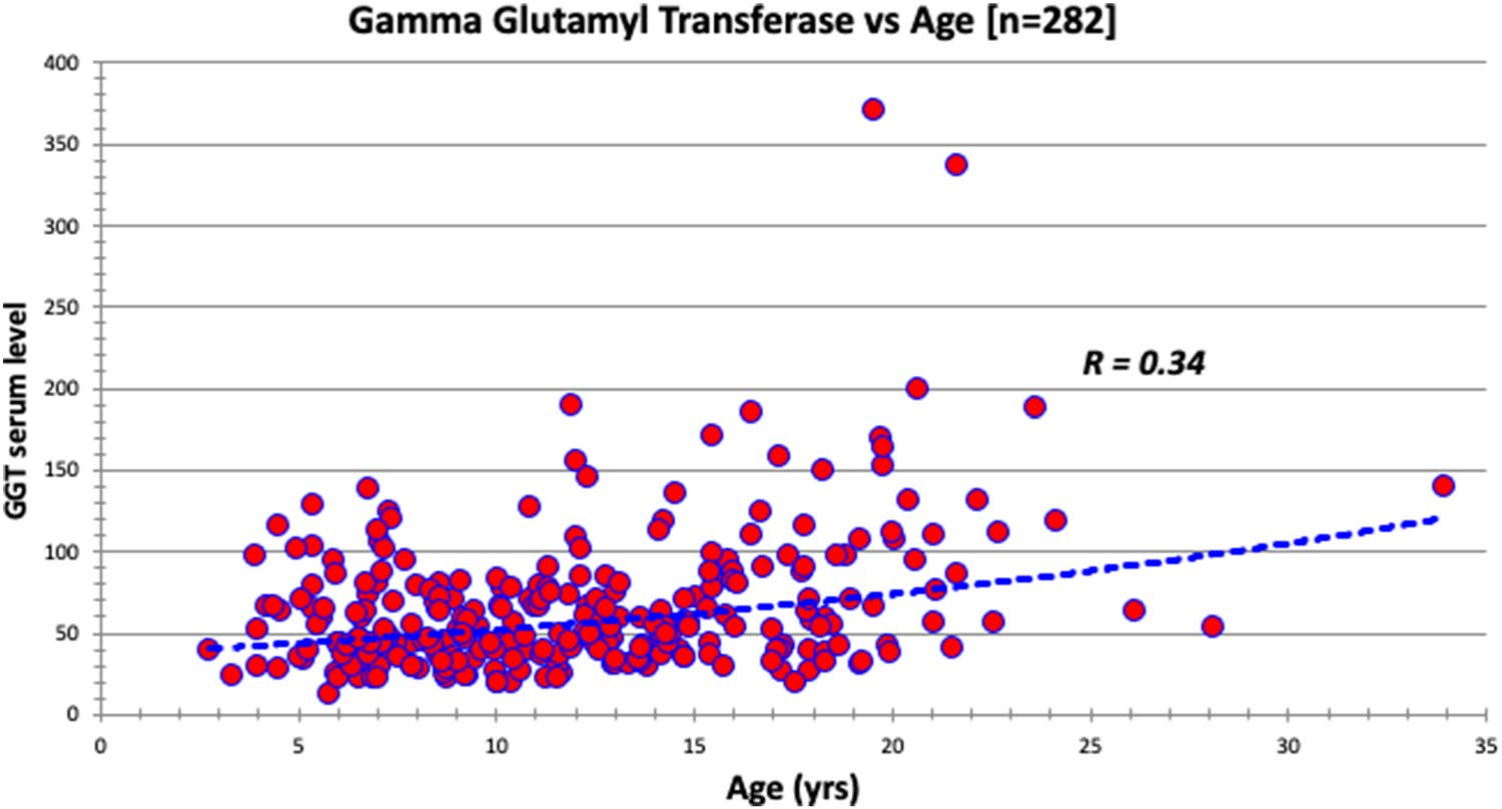
The serum gamma glutamyl transferase (U per liter) distribution based on age at evaluation in a sample of 282 patients evaluated in FORWARD clinic. Upper limit of normal in our laboratory is 21 u/L. The curve is significant at *p* value < 0.01

There are several endeavors and projects that have been engendered and motivated by our experiences in FORWARD to date. We are continuously refining our model of care, now offering targeted focused encounters for patients with specific problems and follow-up needs, as well as altering our testing regimens with an eye towards emerging data on imaging and novel biomarkers. Sharing experiences and the knowledge gained is important for the development of additional Fontan specialty clinics around the world. With over 600 patients seen and over 200 cardiac catheterization and liver biopsies performed at our program, there are important findings and a wealth of information to share concerning this unique population. Examples of information we have begun to analyze in a subset series of our FORWARD patients, include medication burden (Fig. 5) and age-related differences in some potential biomarkers (Figs. 6, 7, 8).

Creating educational tools for families and age-specific tools for patients is essential in teaching the community about the uniqueness of the Fontan circulation. Some of the medical content is complex and often difficult to explain and for patients to grasp. Utilizing technology to develop materials such as video animations can assist with explanations. Patients and families have a strong desire to improve their understanding, which empowers awareness and, thus, offers the potential for best outcomes. Technologies may also be harnessed to educate and encourage adherence to exercise regimens and medication usage through APPs and wearable devices. Our FORWARD program is focused on the child and adolescent, yet with continued success, there is a growing population of adults who will similarly benefit from these multidisciplinary services. Standardized models of care for the adult patient with a Fontan circulation are needed. Finally, multi-institutional efforts to collect large-scale data elements that characterize the life-span trajectory of those with a Fontan circulation is essential and is currently underway. The Fontan Outcomes Network is a national multicenter registry and learning network launched with the aims of collecting data and improving physical, neurodevelopmental, and emotional health in individuals with Fontan circulation (https://www.fontanoutcomesnetwork.org/) [47]. Strengthening of partnerships between patients, practitioners, and institutions can provide hope to fulfilling the promise of creating a path forward for a normal quality and duration of life to individuals with single-ventricle and Fontan circulation.
