# Correction to: A Path FORWARD: Development of a Comprehensive Multidisciplinary Clinic to Create Health and Wellness for the Child and Adolescent with a Fontan Circulation

**DOI:** 10.1007/s00246-022-02952-7

**Published:** 2022-06-13

**Authors:** Jack Rychik, David J. Goldberg, Elizabeth Rand, Edna E. Mancilla, Jennifer Heimall, Nicholas Seivert, Danielle Campbell, Shannon O’Malley, Kathryn M. Dodds

**Affiliations:** 1 Division of Cardiology, Children’s Hospital of Philadelphia, Philadelphia, USA; 2 Division of Gastroenterology, Hepatology and Nutrition, Children’s Hospital of Philadelphia, Philadelphia, USA; 3 Division of Endocrinology and Diabetes, Children’s Hospital of Philadelphia, Philadelphia, USA; 4 Division of Allergy and Immunology, Children’s Hospital of Philadelphia, Philadelphia, USA; 5 Department of Child and Adolescent Psychiatry, and Behavioral Sciences, Children’s Hospital of Philadelphia, Philadelphia, USA; 6 Clinical Nutrition, Children’s Hospital of Philadelphia, Philadelphia, USA; 7 Department of Pediatrics, Perelman School of Medicine at the University of Pennsylvania, Philadelphia, USA; 8 Department of Psychiatry, Perelman School of Medicine at the University of Pennsylvania, Philadelphia, USA; 9 Department of Nursing at the University of Pennsylvania, Philadelphia, USA; 10 Fontan FORWARD Program, Cardiac Center at the Children’s Hospital of Philadelphia, 3401 Civic Center Boulevard, Philadelphia, PA 19104 USA

## Correction to: Pediatric Cardiology 10.1007/s00246-022-02930-z

The conflict of interest statement is missed to include in the original article.

The conflict of interest statement is given below:

Dr. Goldberg is a named inventor on method of use patents regarding the use of udenafil in patients who have undergone Fontan palliation.

Dr. Rychik is a consultant for Mezzion Pharmaceuticals, a company in search of solutions for challenges in the single ventricle population.

The original article has been corrected.

